# Race or Resource? BMI, Race, and Other Social Factors as Risk Factors for Interlimb Differences among Overweight Breast Cancer Survivors with Lymphedema

**DOI:** 10.1155/2016/8241710

**Published:** 2016-06-28

**Authors:** Lorraine T. Dean, Anagha Kumar, Taehoon Kim, Matthew Herling, Justin C. Brown, Zi Zhang, Margaret Evangelisti, Renata Hackley, Jiyoung Kim, Andrea Cheville, Andrea B. Troxel, J. Sanford Schwartz, Kathryn H. Schmitz

**Affiliations:** ^1^Johns Hopkins Bloomberg School of Public Health, Baltimore, MD 21205, USA; ^2^MedStar Health Research Institute and MedStar Georgetown University Hospital, Division of Biostatistics, Washington, DC 20007, USA; ^3^School of Arts & Sciences, University of Pennsylvania, Philadelphia, PA 19104, USA; ^4^The Wharton School of the University of Pennsylvania, Philadelphia, PA 19104, USA; ^5^Perelman School of Medicine, University of Pennsylvania, Philadelphia, PA 19104, USA; ^6^Recruitment, Outcomes, and Assessment Resource Core, University of Pennsylvania, Philadelphia, PA 19104, USA; ^7^The Mayo Clinic, Department of Physical Medicine and Rehabilitation, Rochester, MN 55905, USA

## Abstract

*Introduction*. High BMI is a risk factor for upper body breast cancer-related lymphedema (BCRL) onset. Black cancer survivors are more likely to have high BMI than White cancer survivors. While observational analyses suggest up to 2.2 times increased risk of BCRL onset for Black breast cancer survivors, no studies have explored race or other social factors that may affect BCRL severity, operationalized by interlimb volume difference (ILD).* Materials and Methods*. ILD was measured by perometry for 296 overweight (25 > BMI < 50) Black (*n* = 102) or White (*n* = 194) breast cancer survivors (>6 months from treatment) in the WISER Survivor trial. Multivariable linear regression examined associations between social and physical factors and ILD.* Results*. Neither Black race (−0.26, *p* = 0.89) nor BMI (0.22, *p* = 0.10) was associated with ILD. Attending college (−4.89, *p* = 0.03) was the strongest factor associated with ILD, followed by having more lymph nodes removed (4.75, *p* = 0.01), >25% BCRL care adherence (4.10, *p* = 0.01), and years since treatment (0.55, *p* < 0.001).* Discussion*. Neither race nor BMI was associated with ILD among overweight cancer survivors. Education, a proxy for resource level, was the strongest factor associated with greater ILD. Tailoring physical activity and weight loss interventions designed to address BCRL severity by resource rather than race should be considered.

## 1. Introduction

Obesity is a major risk factor for onset of upper body breast cancer-related lymphedema (BCRL) [1–3], which is a persistent adverse outcome of cancer treatment that affects the physical health and quality of life of up to 35% of the 2.9 million breast cancer survivors in the US [4, 5]. BCRL is an inflammatory condition [6] that arises when removal of lymph nodes during cancer treatment causes lymphatic fluid to build up in the arms, breast, and torso [7]. Because of arm swelling and altered lymphatic function, upper body BCRL may affect a breast cancer survivor's ability to complete activities of daily living and maintain employment and may lead to psychosocial distress or secondary comorbidities [8–11]. In addition to elevated BMI, other known risk factors of BCRL onset include older age, greater number of lymph nodes removed via axillary lymph node dissection, adjuvant radiation therapy [1, 12], and low physical activity [2]. Many of these risk factors are differentially distributed by race, which may explain race differences in BCRL onset. While observational studies have suggested that Black breast cancer survivors are nearly 2.2 times more likely to develop incident BCRL than White breast cancer survivors [13–17] and are also more likely to face greater upper arm disability [18], there has been less exploration of differences in BCRL severity by race. Volume differences between arms, or interlimb difference, are a commonly used objective measure of BCRL severity [19, 20] and are relevant to understanding the impact of the condition. Greater arm volume has been associated with limitations in activity, overall declines in physical functioning, and greater levels of depression [21]. Increasing physical activity and weight loss have been the focus of interventions designed to prevent, stabilize, or reduce BCRL severity [1, 5, 22].

Race is a social construct [23, 24] and racial differences in BCRL severity may represent a litany of embodied social factors. In the case of Black Americans, race may be an indicator of differences in types of treatment received and healthcare access [25] that could translate to differences in BCRL severity or access to lymphedema management resources, such as compression garments or lymphedema therapy sessions. For example, Black breast cancer survivors, on average, have higher BMIs than White breast cancer survivors [26, 27] and are less likely to meet physical activity guidelines [28], both of which could contribute to race-based disparities in BCRL outcomes [29]. Black women are more likely than White women to undergo axillary lymph node dissection, which is associated with greater morbidity than the less invasive sentinel lymph node biopsy [30–33]. While this may be explained in part by the higher likelihood of being diagnosed with more aggressive tumors, there is evidence that even when adjusting for stage and grade of tumors, Black women are more likely to undergo axillary lymph node dissection [34, 35], putting Black women at greater risk of BCRL.

Identifying a profile for risk of greater BCRL severity is recognized as a great need in research [2, 5, 15, 36], and the social risk factors for increased severity can inform both risk profiles and interventions designed to reduce arm volume [13–17]. Social risk factors for increased severity might include indicators of socioeconomic position, such as education or income, which may be linked to access to knowledge of BCRL; access to healthcare resources for BCRL management, such as formal prescription for BCRL treatment; or inflammation-related factors like psychosocial stress due to work obligations or household composition. This cross-sectional observational study uses baseline data from an in-progress clinical trial to explore racial differences in BCRL severity as measured by interlimb volume differences in arm swelling, while accounting for other social, physical, and treatment-related BCRL risk factors, among Black and White [34] breast cancer survivors.

## 2. Materials and Methods

The study population is drawn from 351 women enrolled in the ongoing Women in Steady Exercise Research (WISER) Survivor trial conducted within the University of Pennsylvania's Transdisciplinary Research on Energetics and Cancer (TREC) Center. The WISER Survivor trial was approved by the University of Pennsylvania Institutional Review Board and all participants provided informed consent. WISER Survivor is a one-year randomized controlled weight loss and exercise intervention trial in sedentary overweight breast cancer survivors with breast cancer-related lymphedema (BCRL; see study NCT01515124 at clinicaltrials.gov for details). Participants were recruited from southeastern PA and NJ zip codes, first through letters sent to patients diagnosed with breast cancer from 1999 to 2014 listed in each state's cancer registry or regional hospital-based cancer registry records and then through community events for breast cancer survivors.

Eligibility requirements included (1) having stable (no cellulitis or flare-ups within the past 3 months) breast cancer-related lymphedema according to a standardized clinical evaluation by a Certified Lymphedema Therapist [37] and interlimb arm volume difference of >5% based on the US National Cancer Institute's Common Terminology Criteria for Adverse Events (CTCAE) v3.0; (2) being currently cancer-free at least 6 months after treatment for breast cancer; (3) being overweight or obese (25 ≥ BMI ≤ 50); and (4) being able to walk unaided for at least 6 minutes. Exclusion criteria included (1) medical conditions or medications that would prohibit participation in an exercise program; (2) plans for additional (e.g., curative or reconstructive) surgery during the study period; (3) self-report of weight-lifting within the past year; (4) current engagement in 3 or more times weekly aerobic activity of moderate intensity; (5) plans to move away from the area over the next year; (6) current use of weight loss medication (OTC or prescription); (7) self-report of alcohol or substance abuse within the past 12 months, including at-risk drinking (current consumption of more than 14 alcoholic drinks per week); (8) unstable weight, assessed as weight loss >10 lb in the past 3 months; and (9) a history of bariatric surgery. While women of all racial/ethnic groups were allowed in the study, 17 participants who did not self-report as Black or White (~5% of participants) were excluded from this analysis of racial variation between the two groups. Thirty-six women with bilateral BCRL were also excluded from analysis, plus one woman with a BMI that was borderline for meeting study eligibility.

### 2.1. Primary Outcome

Interlimb arm volume difference was measured by perometry, operationalized as the difference between the left and right arms [36]. The Optoelectronic Perometer® (Juzo USA, Cuyahoga Falls, OH) is a reliable and valid tool to assess interlimb dimension and volume [38–40]. The perometer uses infrared lasers to calculate the total limb length and circumference of each arm based on cross-sectional imaging of the upper limbs and yields girth and volume, as well as the percent difference between affected (side of breast cancer surgery) and unaffected arm limbs. Only assessments taken before randomization were used in this analysis, at which time each participant recorded self-reported arm dominance. To ensure that BCRL self-care practices did not interfere with accurate arm volume measurements, participants were instructed to remove lymphedema compression garments, tape, or bandages at least 1 hour prior to perometry measurement. Larger differences in interlimb volume are significantly positively correlated with greater arm symptom burden and pain [41]. The dominant left or right arm may be favored for use in day-to-day activities so the dominant arm may be larger by volume prior to the development of BCRL or may influence measurement and diagnostic precision of arm volume measurement [42]. Measures of interlimb volume suggest a 3.3% difference in dominant versus nondominant arms in healthy normative populations [43]. Thus, this analysis reduced the volume of the dominant 3.3% before interlimb difference was calculated to account for arm dominance.

### 2.2. Covariates: Demographic, Physical, and Treatment Factors

The demographic, physical, and clinical data were collected via self-report survey at study entry and included race, age, education level (nonoverlapping categories), employment status, total number of persons living in the household, presence of dependent children at home, years since breast cancer diagnosis, type of breast cancer treatment(s), and history of BCRL treatment. Education level cut-offs were based on previous examinations of education, self-rated health, and obesity, which use four categories (less than high school diploma, high school graduate, some college, and college graduate). However, due to sample size, the categories of less than high school and high school graduate were combined, resulting in a 3-category education variable [44]. BMI was determined by objectively measured height and weight using a scale mounted stadiometer and digital scale. Types of breast cancer treatment (chemotherapy, radiation, and hormonal therapies), number of lymph nodes removed, breast cancer stage at diagnosis, and years since breast cancer diagnosis were confirmed by pathology report, when available. If pathology reports were unavailable, the Pennsylvania State Cancer Registry was used to ascertain clinical characteristics. Lymph node removal was later classified as sentinel lymph node biopsy for <5 nodes removed or axillary lymph node dissection or ≥5 nodes removed. Adherence to lymphedema treatment was assessed by self-report [45, 46] and was coded as “yes” if the participant indicated being prescribed and also using one or more of the following treatments at least 25% of the time during the past 3 months: self-care techniques, therapist care, compression garments, bandaging, elevation, skin care, taping, medications, or any other lymphedema treatment in the past 3 months. This variable was coded as “no” if the participant had not been prescribed any of the aforementioned forms of care in the past 3 months or used them less than 25% of the time in the past 3 months. Over 98% of participants reported having health insurance so insurance status was not included in the analysis, due to lack of variation in this covariate.

### 2.3. Statistical Analysis

Data analyses for this cross-sectional observational study were conducted using R version 3.2.2 (R Core Team (2015).* R: A Language and Environment for Statistical Computing*. R Foundation for Statistical Computing, Vienna, Austria. URL https://www.R-project.org/). Missing data on cancer stage of diagnosis and bilateral BCRL were imputed via iterative regression. Multivariable linear regression models were constructed with an outcome of percent change in continuous interlimb volume difference, as a measure of BCRL severity. Models including a covariate for hand dominance [45] were explored. Statistical significance was assessed at *p* < 0.05.

## 3. Results and Discussion

### 3.1. Results

Table 1 shows descriptive statistics for the 296 women in the final analytic sample, of whom 102 were Black (34.5%) and 194 were White (65.5%). Study participants had an average interlimb volume difference of 6.86% (SD = 14.1), corresponding to Grade 1 breast cancer-related lymphedema (BCRL) according to NCI Common Terminology Criteria for Adverse Events (CTCAE) v3.0. The average participant had a body mass index (BMI) of 34, was 60 years old, was the recipient of a college degree or more (47%), was not retired (68%), worked an average of 26 hours per week outside of the home (regardless of retirement status), had 3 people living in the household, and had no dependent children in the home (60.5%). Participants received cancer treatment an average of 8 years from time of study entry, with nearly 70% diagnosed at Stage II or earlier. The majority had axillary lymph node dissection (≥5 lymph nodes removed; 72%), adjuvant radiation (82%), chemotherapy (83%), hormone therapy (57%), and no breast reconstruction (60%). Just under half (46%) had used some form of BCRL treatment at least 25% of the time in the 3 months leading up to their BCRL measurement. There was no significant difference in Black-White racial distribution across educational categories in this study (*p* = 0.37); however, Black women had significantly higher BMI than White women (35.0 versus 33.3; *p* = 0.01).

In Table 2 multivariable linear regression controlling for demographic, clinical, and treatment factors, neither race (−0.26, *p* = 0.89) nor BMI (0.22, *p* = 0.10) was associated with interlimb volume difference. Completing some time in college was associated with a 4.9% smaller interlimb volume difference (−4.86, *p* = 0.03) though being a college graduate was not significantly different compared to completing high school or less (*p* = 0.28). Each year that passed since completing cancer treatment was associated with a 0.55% higher interlimb difference (*p* < 0.001), and receipt of axillary lymph node dissection was associated with an increased 4.75% interlimb difference when compared to sentinel lymph node biopsy (*p* = 0.01). Receipt of or adherence to lymphedema care treatment (4.10, *p* = 0.01) was significantly associated with greater interlimb difference. Models (not presented) including a covariate for arm dominance did not change results.

### 3.2. Discussion

This analysis of breast cancer-related lymphedema (BCRL) in overweight Black and White women found no differences in interlimb arm volume by race or BMI but found large differences by education, suggesting a greater role for resource than race for BCRL severity. Interlimb volume difference is a measure of BCRL severity used by previous researchers and as part of clinical criteria [19, 20]. The strongest factor associated with interlimb difference was educational attainment. Having achieved more than a high school education was associated with a nearly 5 percentage point reduction in BCRL severity as measured by interlimb volume, which represented a stronger influence on BCRL severity than any other physical, clinical, or social risk factor.

The lack of difference in interlimb arm volume by race may mirror findings from a growing number of studies showing that the effects of race on BCRL onset are adjusted away in multivariable analysis [15] and that the observed differences by race may be explained by other social and physical factors, including BMI. While weight gain after cancer treatment has been shown to be a predictor of BCRL onset [7] and increases in arm swelling are associated with BMI ≥ 25 compared to those with BMI < 25 [12], higher BMI was not associated with additional interlimb volume differences in this sample of weight-stable women with BMI ≥ 25. Large weight fluctuations after cancer surgery may increase risk of lymphedema onset [47], but all of the women in the sample had BCRL at the outset and were weight stable at the time of enrollment. This is consistent with the possibility of a threshold for obesity in BCRL severity. For those already classified as overweight or obese, actual BMI may not further exacerbate interlimb volume differences after triggering initial onset.

Previous explorations of BCRL development have focused on the same clinical and physical risk factors that predispose certain populations to greater BCRL onset, such as node dissection type and BMI [48], but have neglected social factors. The present study's results support that physical factors are still valid, as the use of axillary lymph node dissection [1, 36] was significantly associated with an increased interlimb volume difference, and that social factors should have greater consideration. The present analysis is among the first to suggest an influential role for low educational attainment as an important risk factor for increased BCRL severity, and in this case, educational attainment had a stronger association than years since treatment and adherence to BCRL care. Findings of differences by education and not race could not be attributed to the differential distribution of education by race. Few studies have included any extensive social factors, leaving a gap in studies to which we might compare our results. One study of BCRL symptoms adjusting for race and other social factors, which did not show any effect of educational attainment [49], focused on the presence of BCRL, but not severity. This may suggest that the factors related to onset may be different than those associated with severity and progression. A separate study in Hong Kong on interlimb arm volume differences [50] did include a range of social factors related to socioeconomic position, including education and type of job. While that study similarly found no associations between work status and BCRL severity, it also did not find that education played a role. This difference in findings might be ascribed to variations in the economic and social returns of educational attainment [51] in Hong Kong compared with the US. In this paper's findings, the lack of significant difference in interlimb volume when comparing college graduates to only high school degrees may further highlight the variability in economic and social returns at each level of education.

In the United States, educational attainment is considered to be a proxy measure for the larger construct of socioeconomic position, with low attainment being highly correlated with low income, high poverty risk [52], and reduced access to healthcare resources [53]. Potential mediators of BCRL progression among less educated participants remain speculative but candidates include (1) higher levels of hand use due to manual labor job, (2) inflammatory response induced by socioeconomic stressors, (3) greater use of axillary lymph node dissection for low socioeconomic position patients, and (4) low access to resources for BCRL management. However, the first three mechanisms were not supported by our data or previous study data. Hand use has had mixed results [50] and work status did not support differences in interlimb differences. While there were no direct measures of socioeconomic stressors via income or poverty, related household composition measures of number in household and dependent children were not associated with interlimb difference. Receipt of axillary lymph node dissection was also not significantly different by education level. Given our data, the most plausible explanation is that lower educational attainment may be linked to greater interlimb differences through worse access to health resources to manage BCRL.

Low-resource women may not have what they need to navigate worsening BCRL, especially in light of other competing demands that they face in day-to-day life. Still, in line with other studies with similar populations, adherence to BCRL care did not eliminate the interlimb volume disparity [45, 46]. This may suggest that adherence to or usage of BCRL care may not be sufficient to overcome disparities in severity. In other words, underlying social determinants must be addressed in order to reduce disparities in BCRL severity. Poor access to healthcare resources may mean that low-education women are more likely to deal with healthcare providers who are uninformed about BCRL. Thus, they may not receive proper health education to prevent arm swelling from becoming more severe [54]. Proposed educational interventions have been focused on educating patients about BCRL specifically [36] but have not modeled patient overall educational attainment [46, 55–57]. The present results suggest that focusing on adherence to BCRL care [45] and education about BCRL may not be enough to mitigate the risk of greater severity. While there is no evidence that increasing educational attainment itself can reduce the likelihood of BCRL progression or severity, interventions designed to reduce BCRL arm swelling should specially focus on meeting the needs of low-education patients.

Contrary to some studies that have shown that BCRL can dissipate on its own in the first 18–24 months after treatment [2, 58], our analysis aligns with studies suggesting that BCRL may get progressively more severe over time [59, 60]. Higher BCRL severity was associated with greater length of time since cancer treatment. This may support the notion that BCRL is a slowly progressing chronic illness that may worsen with time without active treatment [4]. Adherence to BCRL treatment was associated with higher interlimb volume differences, which may be because women with the most severe BCRL are most likely to be prescribed a treatment regimen and be more likely to adhere to it. Given the potential of increased interlimb volume over time, behavioral interventions could be employed to address progression of arm swelling.

Behavioral interventions to reduce arm swelling have focused on the use of physical activity and weight loss to stabilize or lower BCRL severity [1, 5, 22]. Black breast cancer survivors [13–17], who are at risk for higher levels of obesity [26, 27] and are unlikely to meet recommended physical activity guidelines [28] but who still need to effectively manage BCRL, may benefit from these interventions. Black women in the general population tend to lose less weight than women from other races during behavioral interventions [61], and culturally tailoring interventions improves outcomes only modestly [61, 62]. Unfortunately, thus far, studies on culturally tailored interventions have lacked large sample sizes, long-term follow-up, attention controls, theory-driven behavioral change strategies, or objective measures of physical activity [62]. Nor have these prior studies addressed resource disparities. These interventions may continue to struggle to be successful if they continue to be only tailored culturally and do not account for resource differences among study participants. Since less educated Black women are more prone to have distinct barriers to physical activity [63], there needs to be a reevaluation of how to best tailor physical activity and weight loss interventions focused on reducing arm volume due to BCRL. To effectively address BCRL disparities from onset through progression, interventions addressing BCRL onset could be tailored by race, given previous findings about race differences in onset, while interventions addressing arm volume changes after onset could be tailored by resource level, given findings from the present analysis. Tailoring interventions by resource is a novel approach that may be supported by the results of the present analysis.

While this study boasts a large community-based sample with broad coverage across a metropolitan area, results may not be generalizable, due to the use of a sample of participants who were healthy enough to enroll in a clinical trial. The study sample may be healthier or better-resourced than the overall population of cancer survivors. Nearly half of the study sample had a college education, compared to 29.2% of women over 35 in the US (US Census 2014 estimates), meaning that these women may be more resourced than their peers and that work and household indicators may not represent stressors for the relatively high-resource women who comprised the study population. Since it is not a population-based sample, determining whether or not differences in BCRL severity exist by race or other social factors in the general population is not possible; however, no existing population-based datasets of the general population include information on BCRL severity, making this study the best available sample to assess these relationships. Although perometry is a reliable tool for assessing upper body interlimb volume differences, the use of perometry for measuring interlimb knee [40] and hand volume [64] suggests it may overestimate volume by 2.2 to 7.5% when compared with water volume measurements; however, bias estimates for upper limbs have not been established and this study did not intend to compare differences to water volume-based measures. That said, the average 6.9% interlimb volume difference in this sample exceeds the CTCAE criteria of a 5% interlimb difference indicating BCRL. Differences in interlimb volume may be attributable to morphological differences unrelated to BCRL, which is why this study used both perometry measurements and the examination by the Certified Lymphedema Therapist to confirm that differences in interlimb volume were attributable to BCRL severity.

This dataset was limited to a few proxy measures of socioeconomic position and resource level and did not have data on income or direct measures of psychosocial stress. Education is an imperfect proxy of resource level or socioeconomic position, which may explain the nonmonotonic relationship between education and BCRL severity that has been observed in other analyses of education, obesity, and health outcomes for population-based adult samples [44]. Nevertheless, educational attainment is preferable to other measures of socioeconomic position because it can be determined for all individuals (since not everyone has an occupation or income), and most educational attainment is normally completed by the early adult years, thus avoiding the potential for causation inherent in using income. Future exploration should include direct measures of social status, socioeconomic position, and psychosocial stress.

## 4. Conclusion

This study was designed to explore differences by race in interlimb arm volume differences among breast cancer survivors with breast cancer-related lymphedema (BCRL), accounting for social and physical risk factors for BCRL. In this sample of overweight women, neither race nor BMI was associated with greater interlimb volume, but the social factor of education level had the strongest relationship with interlimb differences. These results suggest a greater role of resource than race for BCRL severity and point to a possible approach to reduce disparities in the burden of BCRL. Interventions focused on reducing interlimb volume may be differentially useful across resource levels. This may stand in contrast to interventions focused on BCRL onset, which may still warrant specific focus on Black women. Future studies should further examine the roles of education level and socioeconomic differences with expanded and more precise measures of education and socioeconomic position.

## Figures and Tables

**Table 1:** Descriptive statistics.

	(*N* = 296)	Mean/*n*	SD/%
Interlimb volume difference^a^ (%)		6.86	14.12
Race	Black	102	34.5%
	White	194	65.5%
Body mass index (kg/m^2^)		33.91	5.94
Age at study entry (yrs)		60	9
Education	High school or less	54	18.2%
	Some college	102	34.5%
	College grad or more	140	47.3%
Retirement status	Not retired	202	68.2%
	Retired	108	32.4%
Hours per week of outside paid work		25.88	18.4
Number of people living in home		3	1
Dependent children at home		117	39.5%
Cancer stage at diagnosis	0	23	7.8%
	I	90	30.4%
	II	90	30.4%
	III	81	27.4%
	IV	12	4.0%
Years since cancer treatment		8.08	5.1
Lymph nodes removed	Sentinel (<5 nodes)	82	27.7%
	Axillary (≥5 nodes)	214	72.3%
Adjuvant radiation therapy		244	82.4%
Adjuvant chemotherapy		246	83.1%
Adjuvant hormone therapy		170	57.4%
Breast reconstruction		118	39.9%
>25% lymphedema care adherence^b^		137	46.3%

^a^Interlimb volume difference represents the volume difference in the arm affected with BCRL and the arm that does not have BCRL; larger values indicate greater BCRL severity.

^b^Lymphedema care adherence was based on the past 3 months and calculated as “no” if the respondent was not being prescribed any treatment or used prescribed treatment less than 25% of the time and “yes” if the respondent used prescribed treatment at least 25% of the time in the past 3 months.

**Table 2:** Multivariable linear regression for interlimb volume difference outcome.

	Coefficient estimate^a^	Standard error	*p*
Race (black ref.)	−0.26	1.79	0.89
Body mass index	0.22	0.14	0.10
Age in years at study entry	0.18	0.12	0.13
*Education: some college versus HS grad or less*	*−4.89*	*2.24*	*0.03*
Education: college graduate versus high grad or less	−2.49	2.31	0.28
Retired	−0.88	2.41	0.72
Hours/week of paid outside work	−0.04	0.06	0.53
Number of people living in home	−0.76	0.66	0.25
Number of dependent children at home	1.34	1.99	0.50
Cancer stage at diagnosis	1.12	0.92	0.23
*Years since cancer treatment*	*0.55*	*0.16*	*<0.001*
*Axillary lymph node dissection*	*4.75*	*1.89*	*0.01*
Radiation therapy	0.04	2.37	0.99
Chemotherapy	−1.45	2.39	0.54
Hormone therapy	−0.44	1.69	0.79
Breast reconstruction	−0.23	1.89	0.91
*>25% lymphedema care adherence*	*4.10*	*1.62*	*0.01*

Italicized items in table represent statistically significant covariates at *p* < 0.05.

^a^Coefficient estimates represent the change in interlimb difference percentage expected with a “yes” response for categorical variables or per unit change for continuous variables.
